# IgE-Crosslinking-Induced Luciferase Expression Test as a Sensitive Indicator of *Anisakis* Allergy

**DOI:** 10.3390/antib14010019

**Published:** 2025-02-25

**Authors:** Haruyo Akiyama, Masashi Niwa, Chisato Kurisaka, Yuto Hamada, Yuma Fukutomi, Reiko Teshima

**Affiliations:** 1 Division of Pharmacotherapeutics, Faculty of Pharmaceutical Sciences, Teikyo Heisei University, Nakano-ku, Tokyo 164-8530, Japan; h.akiyama@thu.ac.jp (H.A.); c.kurisaka@thu.ac.jp (C.K.); 2 Faculty of Veterinary Medicine, Okayama University of Sciences, Imabari 794-8555, Japan; v19m122nm@ous.jp; 3 Clinical Research Center for Allergy and Rheumatology, NHO Sagamihara National Hospital, Sagamihara 252-0392, Japanfukutomi.yuma.da@mail.hosp.go.jp (Y.F.)

**Keywords:** *Anisakis* allergy, anisakiasis, *Anisakis*-specific IgE, luciferase, mast cells, IgE immunoblot

## Abstract

**Background:** *Anisakis* allergy has been increasing, and the diagnosis of it is based on specific serum IgE detection. Recently, the IgE-crosslinking-induced luciferase expression (EXiLE) test has been proposed as convenient tool for detecting functionally specific IgE antibodies. Here, we investigated if the EXiLE test is a useful tool in the diagnosis of *Anisakis* allergy. **Methods:** HuRa-40 cells were sensitized using six serum types from three patients with *Anisakis* allergy at the time of the initial test and after 6–12 months. Thereafter, various concentrations of *Anisakis* worm protein (AWP) were reacted to measure the degree of EXiLE. The degree of EXiLE was compared with *Anisakis*-specific IgE antibody levels measured by the CAP-FEIA method, and the IgE-antibody-binding protein profile was examined using IgE immunoblotting. **Results:** The results showed a good correlation between the CAP-FEIA values and EXiLE obtained with 5 μg/mL of AWP (R = 0.91, *p* < 0.01), a strong response on IgE immunoblotting in the region containing proteins weighing ≥40,000 Da. In addition, after the onset of *Anisakis* allergy, the degree of serum EXiLE decreased in two patients whose *Anisakis*-specific IgE antibody levels decreased over time but increased in one patient whose specific IgE antibodies increased after repeated antigen sensitization. **Conclusions:** Based on these data, the AWP-induced EXiLE test seemed to be useful and convenient for the diagnosis of *Anisakis* allergy, supplementing specific IgE determinants. After allergy onset, the use of this method to observe changes in specific IgE levels over time may be important for predicting the risk of recurrence.

## 1. Introduction

*Anisakis simplex* is a pathogenic nematode belonging to the genus *Anisakis*, family Anisakidae, and order Ascaridida. Human anisakiasis is a gastrointestinal tract parasitic infection caused by third-stage larvae (L3) of *A. simplex.* The life cycle of *Anisakis* nematodes involves marine fish, squid, and crustaceans as intermediate or paratenic hosts and marine mammals as definitive hosts [1]. Humans are accidental hosts and are infected by consuming raw or inadequately cooked fish containing *Anisakis* L3 larvae. Therefore, infection has been directly linked to eating habits.

Symptoms of anisakiasis include severe abdominal pain, nausea, and vomiting caused by damage to the human gastrointestinal tract, primarily the stomach and intestines, secondary to mucosal and submucosal penetration of *Anisakis* L3 larvae [2]. In addition to the pathogenic effects of tissue damage, allergic reactions in sensitized humans can be caused by *Anisakis* larvae. In 1990, the first allergic reaction to *Anisakis*-contaminated fish was reported in Japan by Kasuya et al., who pointed out the allergenic potency of *Anisakis* antigens in addition to active gastrointestinal tract larval penetration [3]. Shortly after that report, 28 patients allergic to *Anisakis* were reported in Spain in 1996 [4].

*Anisakis* allergy is an IgE-mediated allergic reaction primarily caused by consumption of raw seafood containing *A. simplex*, and its recurrence has been thought to be caused by eating raw or undercooked seafood [5,6,7,8,9]. Gastrointestinal allergy is also referred to as gastroallergic anisakiasis (GAA) [10,11], which requires long-term management to deal with the acute phase and prevent prolonged allergic symptoms. In addition, most patients develop *Anisakis* allergy by oral sensitization, although sensitization by inhalation has been reported in occupational allergy [12], in which sensitization is caused by *Anisakis* worm protein (AWP) and not by live *Anisakis* worms. *Anisakis* allergy has been mainly reported in Southeast Asia and Western Europe, with Japan having the highest prevalence (>90%) [13]. Furthermore, in Japan, 10% to 30% of adult allergies are thought to be caused by *Anisakis* [14].

The diagnosis of *Anisakis* allergy is mainly based on measurement of specific IgE level using the CAP-FEIA method, measurement of total IgE level, and skin prick test [14,15]. In addition, the BAT method for measuring specific IgE levels in blood cells has been reported to correlate well with symptoms [16]. Another possible tool is the IgE-crosslinking-induced luciferase expression (EXiLE) test [17,18,19], which is a convenient in vitro method of measuring the function of specific IgE antibodies using cultured cells without using patient blood cells for the diagnosis of *Anisakis* allergy. In this study, we aimed to examine the utility of the EXiLE method for the diagnosis and long-term management of *Anisakis* allergy by comparing it with the CAP-FEIA method and investigating fluctuations in specific IgE blood levels over time in patients detected to have allergic reactions by the EXiLE method.

In particular, we compared the results of the EXiLE method with the specific IgE levels detected by the CAP-FEIA method and evaluated fluctuations in specific IgE blood levels over time in patients detected to have allergic reactions by the EXiLE method.

To the best of our knowledge, this was the first study to demonstrate the application of the EXiLE test.

## 2. Materials and Methods

### 2.1. Preparation of Anisakis Worm Proteins

Species plural identification as *A. simplex* nematodes was performed using a PCR-based method [20]. After being collected from the abdomen of a chub mackerel (*Scomber japonicus)* purchased at local markets in Ehime, Japan, *A. simplex* L3 larvae were washed in PBS and immediately frozen at −20 °C until use. For antigen preparation, according to Carballeda-Sangiao’s method [21], 10 larvae were suspended in 0.8 mL of PBS containing a protease inhibitor cocktail (Sigma-Aldrich, Burlington, MA, USA); ground with biomarker (Nippi Co., Ltd., Tokyo, Japan); and sonicated for 30 s 10 times in an ultrasonic crushing device (BIORUPTORII, Sonic Bio Co., Ltd., Samukawa, Japan). After centrifugation at 12,000× *g* at 4 °C for 5 min, protein extract was obtained and it was used as the antigen sample (i.e., AWP) in the experiment. Protein concentrations were determined using Protein Assay CBB solution (# 29449-44, Nacalai tesque, Kyoto, Japan). The protein solutions were stored in aliquots at −80 °C until use.

### 2.2. Analysis of Serum Samples

This study was approved by the ethics committee of the National Hospital Organization, Sagamihara National Hospital, Okayama University of Sciences, Teikyo Heisei University. Six serum types were collected from three patients during consultation at the first visit and after 6–12 months. The total and *Anisakis*-specific IgE levels in each serum sample were measured using ImmunoCAP-FEIA (Thermo Fisher Scientific, Waltham, MA, USA) [15] at the date of serum collection. The results of the serum sample analyses are presented in Table 1. Pooled serum samples from healthy donors were purchased from Cosmo Bio (Tokyo, Japan) and used as negative controls.

### 2.3. IgE Immunoblotting

First, after adjusting the AWP concentration to 1 mg/mL using PBS, an equal volume of 2× Laemmli Sample Buffer (Bio-Rad, Hercules, CA, USA) and a 1/10 volume of 1M dithiothreitol solution (Nacalai tesque, Kyoto, Japan) were added [22]. After boiling the prepared sample at 100 °C for 2 min, SDS-electrophoresis was performed using 80 μL of sample in a 3.5 cm well and 10 μL of rainbow marker (Cytiva, Uppsala, Sweden) added to a 12% mini protean TGX precast gel (IPG well) (#4861041, Bio-Rad, Hercules, CA, USA) at a constant current of 20 mA. After completion of electrophoresis, the precast gel removed from the fixation plate was applied to a transfer buffer containing 10% methanol (Tris/glycine transfer buffer) for equilibration. The activated 0.2 μm polyvinylidene difluoride membrane (PVDF, Bio-Rad, Herules, CA, USA) was then subjected to overnight electrical transfer at 3 W constant power. After the transfer, the PVDF membrane was immersed in PBS–0.05% Tween 20 (PBS-T), washed under shaking for 5 min, and then immersed in Blocking One (Nacalai tesque, Kyoto, Japan) for 1 h. The cleaned PVDF membrane was cut into 4 mm wide 7 strips. The washed PVDF strips were immersed in primary antibodies from the serum samples of each patient, 10-fold-diluted with 5% Blocking One–PBS-T, and shaken at 4 °C overnight. After incubation with the primary antibody, the strips were rinsed twice with PBS-T and washed three times under shaking for 10 min each. The strips were then immersed in a secondary antibody [goat anti-human IgE, HRP conjugate (Nordic-MUbio, Susteren, Netherlands)], 2000-fold-diluted with 5% Blocking One–PBS-T, and incubated under shaking at room temperature for 1 h. After incubation with the secondary antibody, the strips were rinsed twice with PBS-T and washed three times under shaking for 10 min, and 7 strips were gathered on a Saran wrap sheet and then incubated with Amersham ECL Prime Western Blotting Detection Reagent (Cytiva, Uppsala, Sweden) for approximately 1 min to detect chemiluminescence using an Amersham Imager 680 (Cytiva, Uppsala, Sweden). All experiments were performed at least twice to confirm reproducibility.

### 2.4. Luciferase Assay

HuRa-40 cells were seeded at a density of 5 × 10^4^ cells/50 µL/well in a clear-bottom 96-well plate (#165306, Thermo Fisher Scientific, Waltham, MA, USA) and sensitized overnight using 100-fold-diluted serum samples from the three patients (six types). Subsequently, the cells were washed three times with sterile PBS and then added with different concentrations of AWP solution (0, 0.06, 0.22, 0.78, 2.72, 9.52, 33.3, 117, 408, 1429, and 5000 ng/mL) to make a volume of 50 μL/well. The cells were cultured/stimulated for 3 h at 37 °C in the incubator. We used goat anti-human IgE antibodies (100 ng/mL; Bethyl Laboratories, Montgomery, TX, USA) as positive controls.

After stimulation, a homogeneous luciferase substrate solution (ONE-Glo^TM^ EX, Promega, Madison, WI, USA) was added to a volume of 50 μL/well, and the plate was placed for 5 min at room temperature. The chemiluminescence (luciferase activity) in each well was measured using a GloMax^®^ luminometer (GM3510, Promega, Madison, WI, USA). The EXiLE results were measured as the fold-change in luciferase activity; cell activity without antigen stimulation was set at 1. The cut-off value was established based on the highest value (+3 SD) obtained in the experiments using control serum samples.

### 2.5. Statistical Analysis

The statistical analyses were performed using Microsoft Excel 2016 MSO (version 2412). Spearman’s rank correlation test was performed to compare the results between the EXiLE and CAP-FEIA methods, and the correlation coefficient (R) and *p* value were obtained.

## 3. Results

### 3.1. IgE Immunoblotting

**Figure 1:**
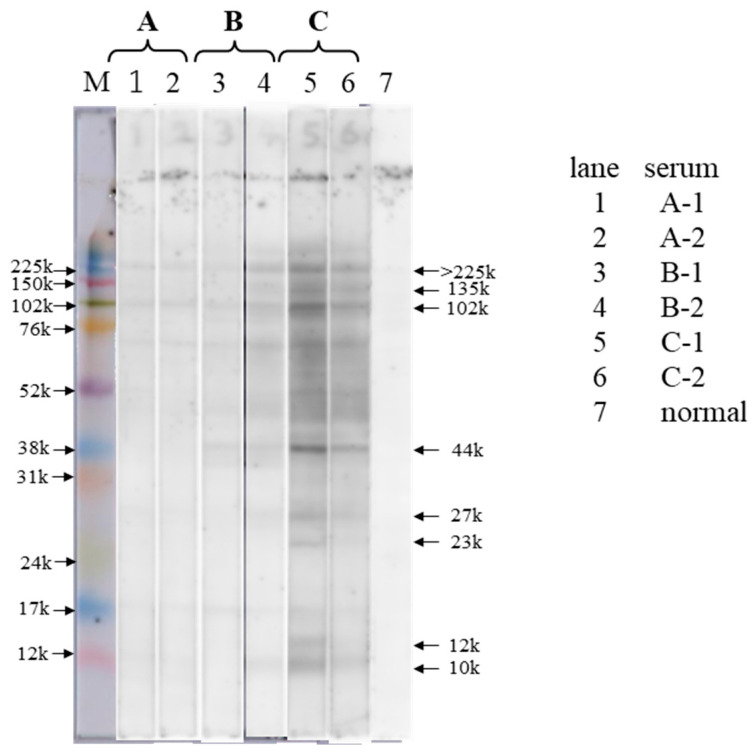
IgE immunoblot analysis of serum samples from patients with *Anisakis* allergy and *Anisakis* worm proteins (AWPs). AWP (10 μg/lane) solution was subjected to 12% SDS-PAGE and IgE immunoblotting using serum samples from patients with *Anisakis* allergy. Lanes 1, 2: patient A’s serum samples after allergy onset (A-1: at 1 y and 6 mo and A-2: at 2 y and 6 mo). Lanes 3, 4: patient B’s serum samples (B-1: at 3 y and 6 mo after allergy onset and B-2: at 1 mo after the second recurrence, which occurred 4 y post-first allergy onset). Lanes 5, 6: patient C’s serum samples after allergy onset (C-1: at 4 mo and C-2: at 10 mo). Lane 7: normal serum samples from a healthy donor. AWP, *Anisakis* worm protein; M, molecular weight marker. Note: The unit “y” means year and “mo” means month.

The results of the IgE immunoblot analyses are presented in Figure 1, and those for the serum sample analyses are shown in Table 1. In patient C, who had high *Anisakis*-specific IgE antibody levels on CAP-FEIA, the serum IgE antibodies were confirmed to be bound to multiple AWPs (Figure 1, lane 5). In particular, according to the identified molecular weight (MW), the major binding proteins seemed to be Ani s 7 (135 kDa) [23], Ani s 7 dimer (>225 kDa), Ani s 2 (102 kDa) [24], and Ani s 3 (44 kDa) [25]. Moreover, the IgE-binding activity of AWP was stronger in serum C-1, which had a CAP-FEIA value of 2400 (Figure 1, lane 5), than in serum C-2, which had a CAP-FEIA value of 87.5 (Figure 1, lane 6). The IgE-binding activity of AWP was weaker in the serum of patient B than in the serum of patient C (Figure 1, lanes 3 and 4). Using serum B-2, which had a CAP-FEIA value of 48.1, a clear IgE immunoblotting pattern was obtained (Figure 1, lane 4), and the main binding proteins had MWs of 135, 102, and 44 kDa. The binding activity in lane 3 using serum B-1 with a CAP-FEIA value of 27.8 was almost the same but weaker than that in lane 4. IgE binding to AWP using the serum samples of patient A was slight (Figure 1, lanes 1 and 2); that in A-1 seemed to be slightly higher than that in A-2 (CAP-FEIA value: 8.06 vs. 6.49).

Based on the results, the main IgE-binding AWPs were considered to have relatively large MWs (>40 kDa), and IgE binding was observed in patients with *Anisakis* allergy and low serum *Anisakis* IgE levels on CAP-FEIA. The intensity of IgE binding seemed to be semiquantitative.

### 3.2. Luciferase Assay

**Figure 2:**
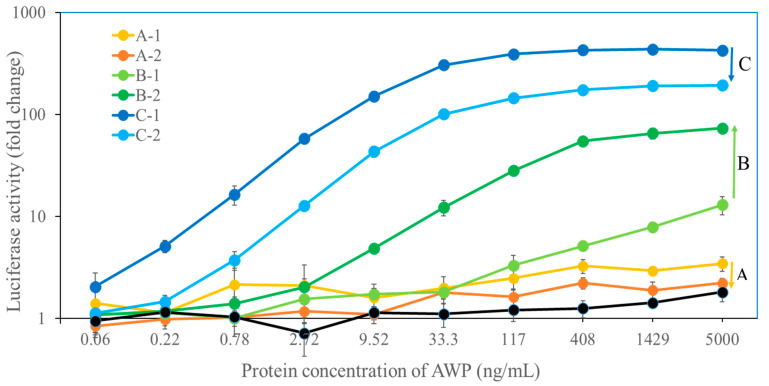
EXiLE test of HuRa-40 cells using serum samples from patients with *Anisakis* allergy and various AWP concentrations. HuRa-40 cells were sensitized overnight with 1:100-diluted serum samples from patients with *Anisakis* allergy or healthy donors and then stimulated with various concentrations of AWP for 3 h. The horizontal axis represents the AWP concentration, and the vertical axis represents the degree of luciferase activity change. The degree of change was calculated as the ratio of the response in the presence of the antigen to the response in the absence of the antigen. Data are presented as mean ± SD (*n* = 3). The changes in luciferase activity over time at AWP concentration of 5000 mg/mL for individual patients were indicated by the arrow on the right side of the figure.

The dose–response curves of the AWP concentration and luciferase activity (fold-change) (Figure 2) showed that the AWP concentration limit differed among the serum samples (0.22 ng/mL for C-1, 0.78 ng/mL for C-2, 9.52 ng/mL for B-2, 117 ng/mL for B-1, and 408 ng/mL for A-1). These results indicated that the detection limit decreased with the increase in the antigen-specific IgE value. Table 2 presents the EXiLE scores in the serum samples. The EXiLE scores in serum samples 1, 2, 3, and 4 corresponded to minimum concentrations of 1429, 117, 9.52, and 0.78 ng/mL, respectively, for the AWP response. We analyzed the correlation between the scores of the CAP-FEIA and EXiLE tests using Spearman’s rank test. There was a good correlation between the CAP-FEIA and EXiLE scores (R = 0.91, *p* < 0.01).

**Figure 3:**
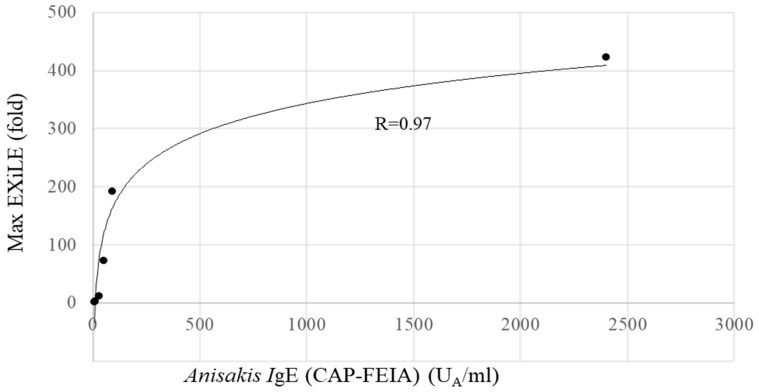
Correlation between Anisakis IgE (CAP-FEIA) and EXiLE in HuRa-40 cells. This figure depicts the results in Table 2 in graphic form through an Excel scatter plot. Logarithmic approximation curve between the CAP-FEIA and EXiLE results obtained with 5 μg/mL of AWP is shown. The R-value was 0.97.

The obtained maximum luciferase activity (EXiLE level) in each serum sample using 5000 ng/mL of AWP demonstrated that as the antigen-specific IgE serum level (CAP-FEIA) increased, the maximum EXiLE level increased, and a good correlation between the two was observed (R = 0.91, *p* < 0.01) according to Spearman’s rank test. Figure 3 shows a scatter plot in Excel 2016 between the CAP–FEIA value and the maximum EXiLE level, and a good correlation was observed. Furthermore, the EXiLE level and CAP-FEIA value obtained with 1429, 408, 117, 33.3, and 9.52 ng/mL of AWP were found to have a good correlation (R > 0.92, *p* < 0.01).

On subsequent examination of the EXiLE level changes over time, the maximum EXiLE level in patients C and A decreased after the initial blood draw (C-1 to C-2 and A-1 to A-2); that in patient B increased (B-1 to B-2) (Figure 2), and this increase was consistent with the increase in the CAP-FEIA value (Table 1). In patient B, the reason for the increase in the maximum EXiLE level over time might be a result of resensitization due to consumption of raw tuna slices before the second blood draw.

## 4. Discussion

*Anisakis* allergy, which has been increasingly observed in recent years, has attracted attention as an IgE-dependent immediate allergy. A high blood titer of *Anisakis*-specific IgE antibody is the key finding for diagnosing *Anisakis* allergy, although results must be carefully interpretated, because some healthy people who have been sensitized do not develop *Anisakis* allergy [16]. In addition, *Anisakis* was reported to have 16 types of allergen components [26], but their clinical significance has not been established. Therefore, several issues in the diagnosis of *Anisakis* allergy remain to be addressed.

Table 3 summarizes the conventional immediate-type allergy diagnosis methods and their possible application to *Anisakis* allergy.

The clinical diagnosis method of immediate allergy is broadly divided into three categories as follows: in vitro studies of Category 3 have the lowest invasiveness, followed by Category 2 ex vivo studies and Category 1 in vivo studies. The most common of these laboratory tests is Category 3 CAP-FEIA (ImmunoCAP) [28,29], which is a simple and highly sensitive method for measuring antigen-specific IgE. CAP-FEIA is also commercially available for *Anisakis* antigens, and although it can be performed normally, it only shows the binding of IgE antibodies to the antigen, and it is not possible to measure biological activity to see if it causes activation of target cells. In addition to CAP-FEIA, the Category 1 skin prick test (SPT) is often used as a screening test for the diagnosis of normal food allergies [28]. The SPT is a method of pricking a small amount of antigen into the skin and looking for redness, and it can be used to measure allergic reactions in the body compared with CAP-FEIA. It has already been used for the diagnosis of *Anisakis* allergy in many cases, but in the case of *Anisakis*, there are restrictions on its application because it is not possible to use commercially available antigens [14,15]. In addition, as a definitive diagnosis of food allergy, an OFC test [27] and a BAT test [28] may be performed to check the relationship with symptoms. Regarding OFC for patients with *Anisakis* allergy, from 1999 to 2004 [30,31,32,33], Spanish researchers reported some studies in which OFC was performed with dead worm proteins, and the challenges were all negative. Therefore, OFC does not seem to be effective for the diagnosis of *Anisakis* allergy. Regarding the BAT test for *Anisakis* allergy [16], one report has been published, and it seems to be a useful technique for diagnosing *Anisakis* allergy. However, as the BAT test requires fresh patient blood for basophil preparation, the test seems to be limited to special laboratories.

In this study, we examined the diagnostic utility of the EXiLE method a simple in vitro measurement of antigen-specific IgE antibodies that reflect clinical symptoms.

Our results showed a good correlation between the EXiLE level and the CAP-FEIA value and a sufficiently low detection limit of the antigen concentration (0.22 ng/mL). The lowest effective *Anisakis* antigen concentration of the EXiLE method was almost the same as the reported lowest effective concentration of peanuts or egg white solution [17,19]. These results strongly suggested that the increased luciferase expression in HuRa-40 cells reflects crosslinking of the antigen-specific IgE bound to FcεR1 on mast cells. Therefore, good EXiLE values were obtained using serum samples of patients with *Anisakis* allergy. Conversely, based on the detection limit of antigen-specific IgE, the sensitivity was slightly lower with the EXiLE method than with the CAP-FEIA method. For example, in serum sample A-2, the CAP-FEIA value was 6.49 U_A_/mL and was judged as positive, whereas the EXiLE value was less than the cut-off and was judged as negative. The reason for the negative ExiLE test result for A-2 was unclear; however, considering that this sample was obtained 2.5 years post-onset, the functional IgE level might have decreased over time. Nevertheless, the EXiLE method appeared to be useful for the diagnosis of *Anisakis* allergy and could supplement other tools, such as the skin prick test, for the determination of specific functional IgE.

In addition, using the EXiLE method in addition to the CAP-FEIA method for long-term management, the reactivity of *Anisakis*-specific IgE was found to change over time after the onset of *Anisakis* allergy. In one patient, the reason for the increase in the maximum EXiLE level over time might be resensitization due to consumption of raw tuna slices before the second blood draw [15]. Long-term monitoring of antigen-specific IgE in serum is important for dietary guidance for patients with *Anisakis* allergy, especially in Japan, where consumption of raw fish is customary. In the future, collecting data from many monitored patients may help prevent the onset and recurrence of *Anisakis* allergy. Moreover, this novel in vitro EXiLE method for *Anisakis* allergy has potential applications for the following: (i) diagnostic supplementation for the determination of serum allergen-specific IgE following the CAP-FEIA test, (ii) screening of allergen components in *Anisakis* worm extract, and (iii) standardization of allergen extracts for clinical use.

## Figures and Tables

**Table 1:** Information on patient sera regarding Anisakis allergy.

Serum Number ^#^	Sex	Age on the Date of Serum Collection	Date of Onset	Date of Serum Collection	Total IgE (IU/mL)	*Anisakis* IgE * (U_A_/mL)	CAP-FEIA Class	Remarks
A-1	M	67	March 2018	October 2019	148	8.06	3	
A-2		67		October 2020	115	6.49	3	
B-1	F	53	March 2016	September 2019	137	27.6	4	allergy symptoms recurred on March 2020.
B-2		53		April 2020	329	48.1	4	
C-1	M	41	March 2019	July 2019	5040	2400	6	
C-2		41		Jan. 2020	872	87.5	5	

* *Anisakis*-specific IgE was determined by CAP-FEIA (Thermo Fisher Scientific). CAP-FEIA was considered positive if the class was ≥2 (i.e., ≥0.70 U_A_/mL). ^#^ All three patients were diagnosed with gastroallergic anisakiasis (GAA). Patient A had not experienced a recurrence of allergic symptoms for 2.5 years. The value of Anisakis IgE at the onset of allergy was 25.1 and then gradually decreased to 8.06 after 1.5 years (A-1) and to 6.49 after 2.5 years (A-2) from the onset of the disease. Patient B had experienced two recurrences since the onset of the disease in March 2016. The serum used in this study was collected one year after the first recurrence (September 2019, B-1) and one month after the second recurrence by consuming raw tuna slices (March 2020) (April 2020, B-2). The patient had tested positive in a skin prick test after the first relapse [15]. Patient C had not experienced a recurrence in the 10 months since the onset of the disease in March 2019. The serum used in this experiment was collected at 4 months (C-1) and 10 months (C-2) after the onset of symptoms.

**Table 2:** EXiLE in HuRa-40 cells sensitized with Anisakis-allergy-patient sera.

		EXiLE Fold by AWP						
	Serum No.	A-1	A-2	B-1	B-2	C-1	C-2	Normal
AWP (ng/mL)								
0.05		1.41	0.84	1.10	1.08	2.04	1.13	0.94
0.22		1.14	0.98	1.13	1.18	**5.10**	1.46	1.15
0.78		2.14	1.02	1.00	1.39	**16.39**	**3.72**	1.03
2.72		2.10	1.17	1.55	2.02	**57.73**	**12.76**	0.72
9.52		1.59	1.09	1.72	**4.87**	**150.59**	**43.26**	1.13
33.3		1.98	1.78	1.80	**12.21**	**304.23**	**100.30**	1.10
117		2.47	1.62	**3.31**	**28.10**	**390.67**	**144.50**	1.21
408		**3.26**	2.22	**5.14**	**54.83**	**426.67**	**173.91**	1.25
1429		**2.93**	1.88	**7.87**	**65.18**	**435.44**	**190.95**	1.42
5000		**3.44**	2.22	**12.93**	**72.99**	**423.67**	**192.86**	1.81
Max EXiLE ** (fold)		**3.44**	2.22	**12.93**	**72.99**	**435.44**	**192.86**	1.81
EXiLE score *		1	0	2	3	4	4	0
Anti-IgE EXiLE (fold)		22.30	25.34	20.54	52.23	88.56	84.77	16.16

* EXiLE scores varied from 0 to 4 [19]. Scores 1, 2, 3, and 4 mean its EXiLE exceeded 2.85 at AWP concentrations of 1429, 117, 9.52, and 0.78 ng/mL, respectively. A score of 0 means negative. ** The EXiLE test was judged to be positive if maximum EXiLE was more than the cut-off level (2.85). Values greater than the cut-off levels are represented in bold. AWP, *Anisakis* worm proteins; EXiLE, IgE-crosslinking-induced luciferase expression.

**Table 3:** Conventional immediate-type allergy tests and possible application to *Anisakis* allergy.

Category	Method	Principle	Possible Application to *Anisakis* Allergy	Detection of Biological Activity
in vivo	Challenge test [27] (oral food challenge)	Direct challenge of allergen under the supervision of a physician	difficult *	(-)
	Skin prick test [28] (SPT)	Introduction of a tiny amount of allergen into the skin	limited **	+
ex vivo	Basophile activation test (BAT) [28]	Activation of basophils collected from peripheral blood	limited ***	+
in vitro	CAP-FEIA [28,29] (ImmunoCAP)	Detection of allergen-binding IgE in the serum with second antibody	possible (commercially available)	−

* The worm extract used in the test is not commercially available. In addition, the insect body itself cannot be used for testing. There are some reports on the oral challenge test using dead *Anisakis* larvae that have yielded negative results in *Anisakis* allergy patients [30,31,32,33]. ** The worm extracts used in the tests are not commercially available, and the tests are conducted only at the laboratory level [14,15]. *** The worm extracts used in the tests are not commercially available, and the tests are conducted only at the laboratory level [16].

## Data Availability

The raw data supporting the conclusions of this article will be made available by the authors on request.

## Informed Concent Statement

Instead of formal informed consent, patient consent was obtained through an opt-out process by publicly disclosing the study details on the homepage of Sagamihara National Hospital according to the Japanese Act on the Protection of Personal Information (Article 27(2)).

## Abbreviations

CAP-FEIA	CAP–fluorescence enzyme immunoassay
IgE	Immunoglobulin E
HRP	Horseradish peroxidase
PBS	Phosphate-buffered saline
SDS	Sodium dodecyl sulfate
CBB	Coomassie Brilliant Blue
SD	Standard deviation
